# Clinical and kinematic predictors of foot orthoses efficacy in individuals with patellofemoral pain syndrome

**DOI:** 10.1186/1757-1146-4-S1-O5

**Published:** 2011-05-20

**Authors:** Christian J Barton, Pazit Levinger, Kay M Crossley, Hylton B Menz

**Affiliations:** 1 Musculoskeletal Research Centre, Faculty of Health Sciences, La Trobe University, Bundoora, Victoria 3086, Australia; 2 Department of Mechanical Engineering, University of Melbourne, Victoria 3052, Australia

## Background

There is emerging evidence that foot orthoses are effective in the management of patellofemoral pain syndrome (PFPS), however the identification of those most likely to benefit from orthoses has not been adequately explored. The primary aim of this study was to develop a clinical prediction rule to help identify individuals with PFPS who are most likely to benefit from foot orthoses. The secondary aim was to determine whether kinematic measures of lower limb function are associated with foot orthoses efficacy.

## Methods

Sixty individuals with PFPS were issued with non-customised prefabricated foot orthoses (Vasyli Pro, Vasyli International), and patient-reported level of improvement was documented at 12 weeks. Potential baseline predictor variables of interest included demographics, pain characteristics, foot and footwear characteristics, and functional performance measures. In a subset of 26 participants, 3D kinematics of the lower limb were measured using a motion analysis system and these variables were explored for their relationship with the degree of clinical improvement.

## Results

Of the 57 participants who completed the study, 25% (14) reported marked improvement at 12 weeks. The probability of marked improvement was 78% if three out of the following four criteria were met: (i) a footwear motion control properties subscale of less than 5 (indicative of less supportive footwear), (ii) usual pain less than 22mm on a 100mm visual analog scale, (iii) ankle dorsiflexion range of motion with knee flexed less than 41 degrees, and (iv) immediate reduction in single leg squat pain when wearing the orthoses. In the kinematic sub-analysis, only one variable was significantly associated with marked improvement: greater peak rearfoot eversion.

## Conclusions

Individuals with PFPS who wear less supportive footwear, report lower levels of pain, exhibit less ankle dorsiflexion range of motion and who report an immediate reduction in pain with foot orthoses when performing a single leg squat are more likely to benefit from foot orthoses. In addition, the kinematic analysis revealed that foot orthoses may be most effective in individuals with PFPS who demonstrate increased foot pronation during gait.

